# Automatic Detection of Blood Vessels in Retinal Images for Diabetic Retinopathy Diagnosis

**DOI:** 10.1155/2015/419279

**Published:** 2015-02-24

**Authors:** D. Siva Sundhara Raja, S. Vasuki

**Affiliations:** ^1^Department of ECE, SACS MAVMM Engineering College, Madurai, Tamil Nadu 625 301, India; ^2^Department of ECE, Velammal College of Engineering and Technology, Madurai, Tamil Nadu 625 009, India

## Abstract

Diabetic retinopathy (DR) is a leading cause of vision loss in diabetic patients. DR is mainly caused due to the damage of retinal blood vessels in the diabetic patients. It is essential to detect and segment the retinal blood vessels for DR detection and diagnosis, which prevents earlier vision loss in diabetic patients. The computer aided automatic detection and segmentation of blood vessels through the elimination of optic disc (OD) region in retina are proposed in this paper. The OD region is segmented using anisotropic diffusion filter and subsequentially the retinal blood vessels are detected using mathematical binary morphological operations. The proposed methodology is tested on two different publicly available datasets and achieved 93.99% sensitivity, 98.37% specificity, 98.08% accuracy in DRIVE dataset and 93.6% sensitivity, 98.96% specificity, and 95.94% accuracy in STARE dataset, respectively.

## 1. Introduction

**Figure 1:**
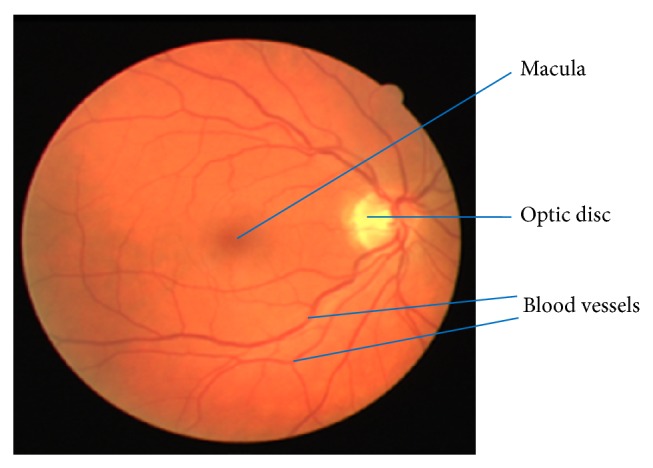
Retinal image showing blood vessels and OD.

All over the world, there are several health disorders affecting people of all ages. One of such most prevalent disorders is type 2 diabetes [1]. People with diabetes are prone to be affected by DR, a retinal disease causing serious loss of vision in diabetic patients. DR causes retinal abnormalities in the form of microaneurysms, hemorrhages, and exudates, which are the various types of disorders caused due to the damage of blood vessels [2, 3]. The retinal blood vessels originate from the center of OD and spreads over the region of the retina, shown in Figure 1. The blood vessels are responsible for supplying the blood throughout the entire region of the retina. The retinal blood vessels are damaged due to the aging of the people and other factors [1]. Microaneurysms, hemorrhages, and exudates lesions are formed in retinal image due to the damage in retinal blood vessels. The retinal blood vessel detection and segmentation are one of the preprocessing steps for the detection and diagnosis of these abnormal lesions. Ignoring these lesion symptoms leads to the loss of vision, as these symptoms are not exposed easily and require diagnosis at an earlier stage. It is essential for the diabetic patients to have their eye checked often to avoid DR. Early screening and diagnosis of DR in diabetes patients reduce the risk of losing the eyesight by 50% [1].

Early detection of DR can be screened by the analysis of blood vessels within the retina [4]. The automatic detection and segmentation of retinal blood vessels are important for the automatic detection of DR [5]. Hence, automatic detection of retinal blood vessel is proposed in this paper. Several computer aided automatic segmentations of retinal blood vessels [4–12] exist whose segmentation methodologies are mainly based on feature computation on pixel variations. These methodologies employed complex pre- and postprocessing steps and larger segmentation time, thereby making the retinal blood vessel detection process more complex. Hence, a simple and efficient computer aided automatic retinal blood vessel detection methodology is proposed in this paper to eliminate such kind of limitations.

In existing methodologies [5, 8–12], the vessel segmentation has been done without eliminating the OD. Since the optic disc is the converging point of all the vessels within the retina, elimination of OD boundary makes some visible blood vessels cross through it. Hence, the boundary of OD has to be removed before the vessels are segmented, as it may lead to misdetection of OD pixels overlapping with the blood vessels. In order to avoid such misdetections, in this paper, we propose an automatic segmentation and elimination of OD boundary and then proceed with the process of vessel segmentation, which further improves the accuracy in vessel detection.

Section 2 presents state-of-the-art methods for the automatic detection of retinal blood vessels in retinal images. Sections 3 and 4 describe the materials and methods used in this paper for the detection of blood vessels.  Section 5 describes the evaluation results for retinal blood vessels in detail using the images available in different datasets. Finally, Section 6 depicts the conclusion.

## 2. Related Works

Mendonça and Campilho [6] have proposed vessels segmentation algorithm using vessel centerlines followed by the vessel filtering process. Multiscale morphological enhancement technique was used to improve the contrast of the blood vessels. The authors achieved 96.33% accuracy in DRIVE dataset and 95.79% accuracy in STARE dataset. Wilcoxon matched pairs testing algorithm was used to prove the accuracy of their results. Palomera-Pérez et al. [4] have used feature extraction based region growing algorithm for the segmentation of blood vessels. The domain partitioning based parallelism was used to group the vessels. The authors achieved 92.5% accuracy in DRIVE dataset and 92.6% accuracy in STARE dataset. Fraz et al. [7] used ensemble classifier to segment the vessels. The gradient vector field and Gabor transform were constructed and used as the feature in ensemble classifier. The authors achieved 72.62% sensitivity, 97.64% specificity, and 95.11% accuracy in STARE dataset and 74.06% sensitivity, 98.07% specificity, and 94.8% accuracy in DRIVE dataset.

Xiao et al. [8] have used spatial constraint based Bayesian classifier for segmenting the blood vessels. The modified level set approach was used to extract the vessel boundaries in the retinal images. The energy function was used to differentiate the vessels from the background pixels. The authors achieved 75.13% sensitivity, 97.92% specificity, and 95.29% accuracy in DRIVE dataset and 71.47% sensitivity, 97.35% specificity, and 94.76% accuracy in STARE dataset. Manoj et al. [9] have used feed forward back propagation neural network classifier for blood vessel segmentation. The features like gradient vector regions, morphological transformation vectors, and line strength vectors were extracted from the retinal images. The 94.29% sensitivity, 98.75% specificity, and 96.23% accuracy were achieved in DRIVE dataset and 93.14% sensitivity, 98.84% specificity, and 95.83% accuracy were achieved in STARE dataset.

Bansal and Dutta [10] have proposed fuzzy algorithm for the segmentation of vessels. The blockwise fuzzy rules were constructed for the classification of vessels and nonvessels. The 86.53% sensitivity, 98.33% specificity, and 97.28% accuracy in DRIVE dataset were achieved in this paper. Marín et al. [5] have proposed grey level and moment feature based supervised classifier for blood vessel segmentation. The background homogenization was applied to improve the contrast of the retinal blood vessels. The authors achieved 70.67% of sensitivity, 98.01% of specificity, and 94.52% of accuracy in DRIVE dataset and 69.44% of sensitivity, 98.19% of specificity, and 95.26% of accuracy in STARE dataset. The total time required to process a single image was approximately one minute and thirty seconds (for both images in DRIVE and STARE database), running on a PC with an Intel Core2Duo CPU at 2.13 GHz and 2 GB of RAM.

Budai et al. [11] have used extended version of Frangi algorithm for the segmentation of blood vessel tree from the retina. The methodology used in this work achieved vessel detection computational time of 1.31 seconds for images in STARE dataset and 1.04 seconds for images in DRIVE dataset. Soares et al. [13] have used 2D-Gabor wavelet transform and supervised classifier for the detection and segmentation of blood vessels in retinal images. The average computational time achieved in this work was approximately 180 seconds for images in both STARE and DRIVE datasets. The proposed method stated in this paper reduced retinal blood vessel segmentation average computational time and increases the average sensitivity, specificity, and accuracy.

## 3. Materials

The two publicly available retinal image datasets such as DRIVE and STARE are used in this paper to analyze the performance of the retinal blood vessel segmentation with respect to ground truth images. These two datasets consist of both normal and abnormal retinal images.

### 3.1. DRIVE Dataset

This dataset [14] is made using 400 diabetic patients aged between 25 and 90 years. 40 images have been randomly selected, out of which 33 do not show any sign of DR and 7 with signs of mild early DR. Each image has been JPEG compressed. The images were acquired using a Canon CR5 nonmydriatic 3CCD camera with a 45° field of view (FOV). Each image was captured using 8 bits per color plane at 768 × 584 pixels. The FOV of each image is circular with a diameter of approximately 540 pixels. For each image, a mask image is provided that delineates the FOV. For this database, the images have been cropped around the FOV.

### 3.2. STARE Dataset

This dataset [15] comprises 20 eye-fundus color images (10 of them contain pathology) captured with a TopCon TRV-50 fundus camera at 35° FOV. The images were digitalized to 700 × 605 pixels. This dataset contains two sets of manual segmentations made by two different observers. Performance is computed with the segmentations of the first observer as ground truth. DRIVE dataset also contains manually blood vessel segmented images.

## 4. Proposed Blood Vessel Segmentation Method

**Figure 2:**
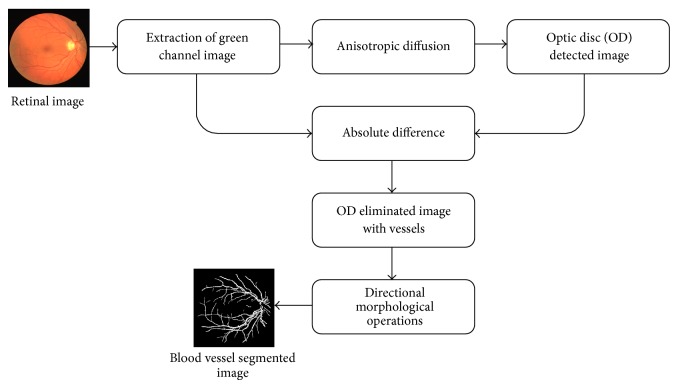
Proposed blood vessel segmentation system.

The proposed system for retinal blood vessel segmentation consists of (1) optic disc detection, (2) optic disc segmentation and elimination, and (3) blood vessels detection and segmentation. The proposed system is illustrated in Figure 2.

The retinal blood vessels originate from the center of OD and spread over the region of the retina. The detection and elimination of OD are important for the segmentation of blood vessels accurately from the retinal images due to the interference between OD and blood vessels.

### 4.1. Optic Disc Segmentation

The retinal image consists of three channels such as green, red, and blue. Among these three channels, red channel is oversaturated and blue channel is underilluminated for blood vessel detection. The green channel is considered for blood vessel detection due to its high contrast between blood vessels and its background. The existence of blood vessels within the optic disc region may cause misdetection of pixels belonging to blood vessels as OD. In order to detect and segment the retinal blood vessels accurately, the optic disc should be detected and eliminated from the retinal image. The anisotropic diffusion filter [16] is used to detect and segment the optic disc from the green channel retinal image.

### 4.2. OD Segmentation by Anisotropic Diffusion Filter

**Figure 3:**
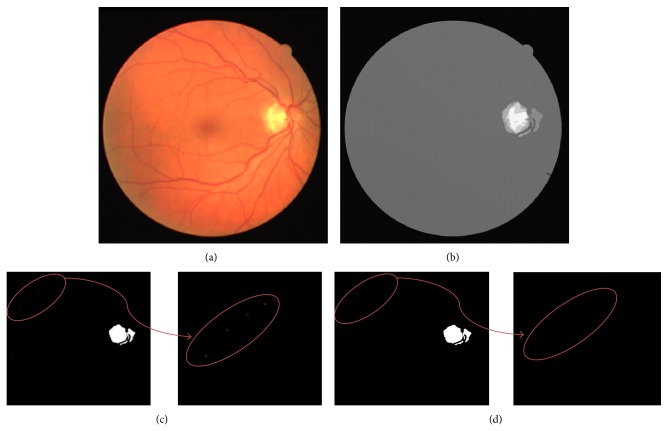
(a) Retinal image from DRIVE dataset, (b) showing its anisotropic diffused image, (c) OD segmented image which includes noisy pixels resembled as OD pixels, and (d) OD segmented image accurately.

The anisotropic diffusion filter is a nonlinear filtering technique employed in the process of segmenting the optic disc. In Perona and Malik [16], anisotropic filter made use of four neighboring pixels in the noise removal process from the images. In this paper, this nonlinear filter is employed over the retinal image taken from DRIVE dataset (Figure 3(a)) to detect the OD boundary accurately. The equation of anisotropic nonlinear diffusion filter involves the calculation of the divergence of the Laplacian and gradient operators of the image.

The discrete anisotropic filter equation is expressed as follows:

(1) Ianiso+1=Ianisom+ψ∫DN·∇NPNi+DS·∇SPSihhihhhh+DE·∇EPEi+DW·∇WPWihhihhhh+DNE·∇NEPNEi+DSE·∇SEPSEihhihhhh+DSW·∇SWPSWihhhhhh+DNW·∇NWPNWi∫,

where *I*_aniso_ is the image to be diffused and the indices N, S, E, W, NE, NW, SE, and SW indicate the neighborhood pixels around the center pixel. The value of *ψ* lies between 0 and 1 as a constant.  Table 1 shows the 3 × 3 anisotropic diffusion mask [16] by making use of eight neighboring pixels.

The Laplacian operator (∇) for eight neighborhood pixels can be estimated using first order derivatives and the center pixel *P*_*i*_ can be expressed as

(2) $$\begin{array}{c} \nabla_{\text{Dir}}\left[P_{\text{Dir}}\right]=P_{\text{Dir}}-P_{i}, \end{array}$$

where “Dir” denotes N, E, S, W, NE, NW, SE, and SW pixels, respectively, and is given as

(3) $$\begin{array}{c} \text{Dir}=\left\{P_{\text{N}},P_{\text{S}},P_{\text{W}},P_{\text{E}},P_{\text{NE}},P_{\text{NW}},P_{\text{SE}},P_{\text{SW}}\right\}. \end{array}$$

The diffusion coefficient “*D*” can be expressed as

(4) $$\begin{array}{c} D_{\text{Dir}}=C\left(\left\|P_{\text{Dir}}\right\|\right). \end{array}$$

The parameter “*C*” is diffusion kappa coefficient and can be expressed as

(5) $$\begin{array}{c} C=\frac{1}{1+{\left(\left\|\nabla P\right\|/k_{a}\right)}^{2}}, \end{array}$$

where *k*_*a*_ is a constant denoting the kappa factor, which controls the sensitivity of the edges in the image. The following constraints should be adopted for determining the kappa factor as

(6) $$\begin{array}{c} \left\|\nabla P\right\|<k_{a};\;\text{for nonedge regions}\\ \left\|\nabla P\right\|\geq k_{a};\;\text{for edge regions}. \end{array}$$

In this paper, anisotropic diffusion filter convolves the “*R*” channel image with its directional filter coefficients [16] and the filtering process is denoted as

(7) $$\begin{array}{c} P_{i}=P_{o}*c_{j}\;\left(i=1,2,\ldots ,8;\;j=1,2,\ldots ,8\right), \end{array}$$

where *P*_*i*_ is the filtered image or diffused image (Figure 3(b)), *P*_*o*_ denotes the “*R*” channel image (OD region is clearly visible in red channel), and *c*_*j*_ denotes the directional filter coefficients.

The eight directional filter coefficient templates [16] are tabulated in Table 2.

The histogram thresholding [3] is now applied over the diffused image (Figure 3(b)) to detect some noisy pixels resembling the OD pixels and is shown in Figure 3(c). The region with noisy pixels is enlarged and is shown in Figure 3(c). These noisy pixels are further removed by morphological multiplication of this image with binary morphological mask (Section 4.3). The final OD segmented image obtained is enlarged and shown in Figure 3(d).

### 4.3. Creation of Binary Morphological Mask

**Figure 4:**
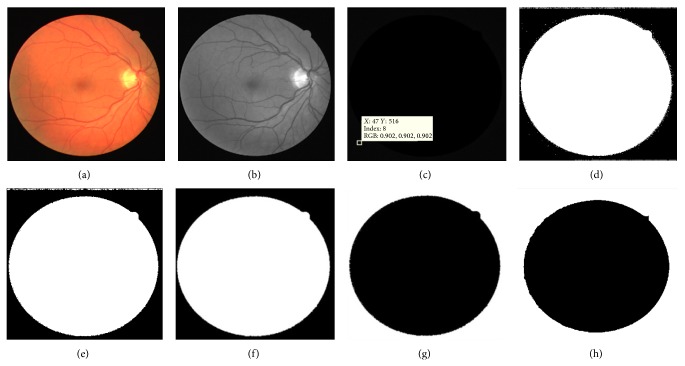
(a) Retinal image, (b) Green channel image, (c) threshold image, (d) inverted binary image with noise, (e) morphologically opened image without noise, (f) border cleared mask image, (g) binary morphological mask image, and (h) dilated binary morphological opened mask image.

**Figure 5:**
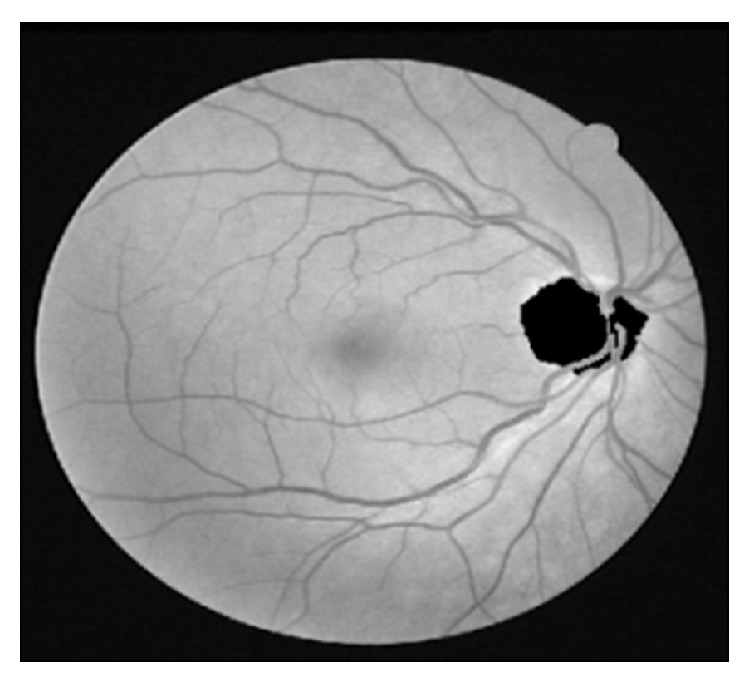
OD eliminated image.

The green channel is extracted from retinal image for the creation of binary morphological mask. The pixels in the green channel image (Figure 4(b)) having the value greater than 10 (this threshold value is determined using thresholding method stated in [3]) are set to zero (Figure 4(c)) and the remaining pixels are having the integer values (different grey level intensities) as in the green channel image. This image is inverted in order to get the binary image (Figure 4(d)). This inverted image gives a mask image of the retina. The morphological opening function with disc shaped structuring element with 1 pixel radius is applied on this binary image to remove the noisy pixels around the retinal image region (Figure 4(e)). Next, suppress the structures that are lighter than their surroundings and that are connected to the image border using 4-connected neighborhood pixels (Figure 4(f)). The binary morphological mask (Figure 4(g)) is created by applying the inversion function on the image. The morphological dilation with disc shaped structuring element with 15-pixel radius is now applied on the binary mask image to sharpen the retinal mask region shown in Figure 4(h). This sharpen mask image is logically multiplied with OD detected image (Figure 3(c)) in order to produce the OD segmented image accurately as shown in Figure 3(d). Now, the OD region is subtracted from the green channel image shown in Figure 5.

**Figure 6:**
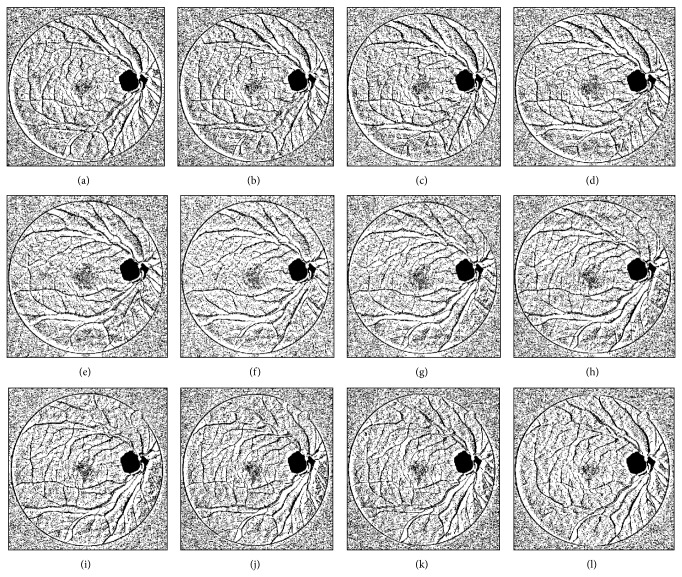
(a) Template at 15°; (b) template at 30°; (c) template at 45°; (d) template at 60°; (e) template at 75°; (f) template at 90°; (g) template at 105°; (h) template at 120°; (i) template at 135°; (j) template at 150°; (k) template at 165°; and (l) template at 180°.

**Figure 7:**
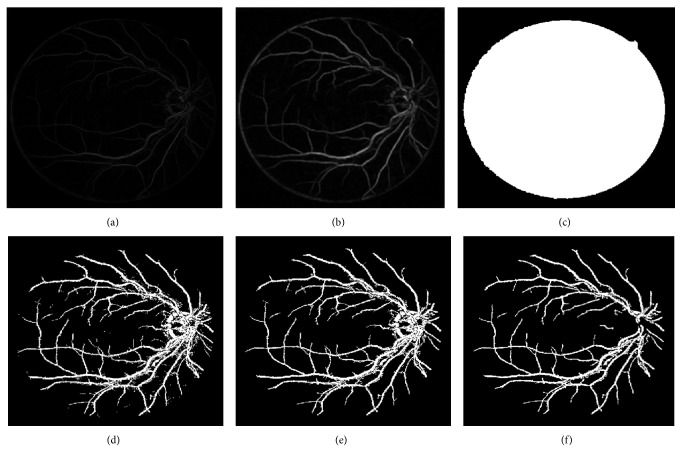
(a) Maximum response image, (b) threshold image, (c) eroded mask image, (d) vessel detected image, (e) noisy pixel removed image having OD, and (f) vessel segmented image.

The blood vessels present in the retinal image can be accurately detected using the templates of different orientations which were illustrated by Soares et al. [13]. Our methodology employs the process of creating 12 templates from OD eliminated image (Figure 5), each having orientation of vessels at 12 different directions, that is, from 15° to 180°, at increments of 15°. Each template analyzes only 10 pixels in line with the direction of its corresponding orientation, thus creating 12-template images (Figures 6(a)–6(l)). These obtained template images are then morphologically opened with the green channel image to obtain another 12 images containing the vessels detected at each orientation. Finally, each vessel pixel detected in these images is combined with other templates in order to obtain the maximum response image (Figure 7(a)) which gives the final accurate vessel detected image.

The thresholding [3] is applied on the maximum response image in order to enhance the blood vessels as shown in Figure 7(b). The thresholding procedure is explained as follows.

(1) Select an initial estimate for *T*0 (initial threshold, say 15, takes any value randomly between 0 and 255).
(2) Segment the maximum response image using *T*0. This will produce two groups of pixels: *M*1 consisting of all pixels with gray level values >*T*0 and *M*2 consisting of pixels with values ≤*T*0.
(3) Compute the average gray level values mean1 and mean2 for the pixels in regions *M*1 and *M*2.
(4) Compute a new threshold value (*T*):
  (8) $$\begin{array}{c} T=\left(\frac{1}{2}\right)*\left(\text{mean}1+\text{mean}2\right). \end{array}$$
(5) Repeat steps (2) through (4) until difference in *T* in successive iterations is smaller than a predefined parameter *T*0.

The binary morphological operations are now applied over the threshold image (*G*). The dilation and erosion are the two fundamental operations in binary mathematical morphology approach. These two operations have two operands, first operand is considered as threshold image and the second operand is its structuring element (*s*) which can be determined by the shape and size of the blood vessel in the retinal image. The operation of dilation followed by erosion over the threshold image is defined by

(9) $$\begin{array}{c} G⊕s=\left\{x\;|\;\left[{\left(\hat{s}\right)}_{x}\cap G\right]\right\},\\ G⊙s=\left\{x\;|\;{\left(s\right)}_{x}\subseteq G\right\}, \end{array}$$

where (*s*)_*x*_ represents the translation of the structuring element (*s*) by the vector “*x*” and $\left(\hat{s}\right)$ represents the symmetry of “*s*.”

In this paper, the structuring element “*s*” is chosen as 15 after applying several iterations. The threshold image is now subtracted from morphologically processed image and described as

(10) $$\begin{array}{c} I_{\text{vessel}}=G-\left\{G-\left(G⊙s\right)\right\}. \end{array}$$

The border cleared mask image (Figure 4(f)) is eroded by disc shape structuring element 10 pixels in order to remove the outer circle in Figure 7(b). This eroded mask image (Figure 7(c)) is morphologically multiplied with threshold image (Figure 7(b)) to get the outer circle cleared and enhanced blood vessel segmented image with noises (Figure 7(d)). Now, these noisy pixels are removed that have fewer than 50 connected component pixels as shown in Figure 7(e). The OD pixels are then removed from this image by multiplying this noise removed image with OD segmented image (Figure 3(d)) in order to get the vessel segmented image accurately (Figure 7(f)).

**Figure 8:**
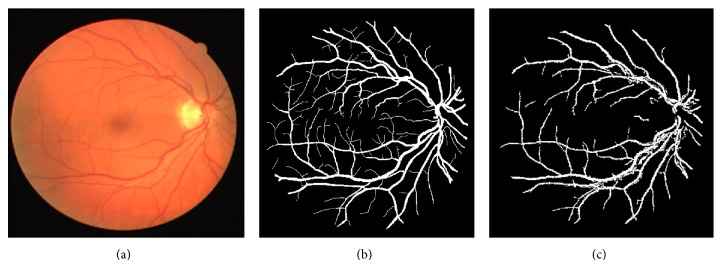
(a) Retinal image from DRIVE dataset; (b) ground truth image; and (c) vessel segmented image by the proposed method.

**Figure 9:**
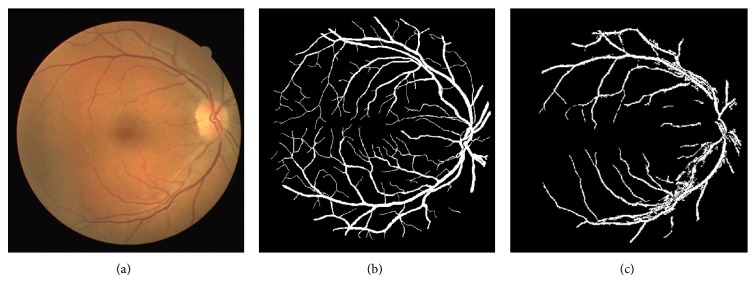
(a) Retinal image from STARE dataset; (b) ground truth image; and (c) vessel segmented image by the proposed method.

Figures 8 and 9 show the blood vessel segmented images by our proposed method, considering the source and ground truth retinal images from DRIVE and STARE datasets, respectively.

## 5. Results and Discussion

### 5.1. Evaluation Details of Blood Vessel Segmentation

The performance of proposed retinal blood vessel segmentation algorithm is tested in two publicly available datasets DRIVE [14] and STARE [15]. These datasets are having manually blood vessel marked images which are called ground truth images. The performance of proposed vessel segmentation results is analyzed with respect to ground truth images. The parameters used in Marín et al. [5] are utilized to measure the performance of the proposed results and they are given in

(11) $$\begin{array}{c} \text{Sensitivity}\left(\text{Se}\right)=\frac{\text{TP}}{\left(\text{TP}+\text{FN}\right)},\\ \text{Specificity}\left(\text{Sp}\right)=\frac{\text{TN}}{\left(\text{TN}+\text{FP}\right)},\\ \text{Accuracy}\left(\text{Acc}\right)=\frac{\left(\text{TP}+\text{TN}\right)}{\left(\text{TP}+\text{FN}+\text{TN}+\text{FP}\right)}. \end{array}$$

Sensitivity is defined as the ratio between the number of TP and the sum of the total number of TP plus FN. The value of sensitivity lies between 0 and 1. Specificity is defined as the ratio between the number of TN and the sum of the total number of FP plus TN. The value of specificity also lies between 0 and 1. The parameter sensitivity and specificity are calculated with respect to ground truth images available in the corresponding datasets. The value of Se and Sp should be high for better vessel segmentation results. The true positive refers to the correctly detected blood vessels, true negative refers to the wrongly detected blood vessels, and false positive and false negative refer to the correctly and wrongly detected nonblood vessel pixels.

Tables 3 and 4 summarize the results of this proposed work in both DRIVE and STARE databases, respectively. These tables show the performance of the proposed method for all the images in the DRIVE (33 normal and 7 abnormal images) and STARE (10 normal and 10 abnormal images) datasets. The proposed algorithm detects and segments the retinal blood vessels at an average sensitivity rate of 94.35% and specificity rate of 98.65% and accuracy rate of 98.24%, respectively, in DRIVE dataset. The results obtained are compared with other state of the art [4–11] in DRIVE dataset and tabulated in Table 5.

The proposed algorithm detects and segments the retinal blood vessels at an average sensitivity rate of 94.1% and specificity rate of 98.98% and accuracy rate of 95.94%, respectively, in STARE dataset. The obtained results are compared with other state of the art [4–9, 11] in STARE dataset and tabulated in Table 6. The comparison of proposed method is done with other similar vessel detection methods which did not eliminate OD but considered the vessel segmentation alone.

The retinal blood vessel average computational time of the proposed methodology and the methods presented in the state of the art [5, 11, 13, 17] is compared and stated in Table 7 for both STARE and DRIVE database retinal images. The proposed method has reduced average retinal blood vessel computational time by 3.6% in STARE database and 2.98% in DRIVE database when compared with methodology stated in [11].

The proposed vessel segmentation algorithm works well in both dataset retinal images and produced satisfactory results in terms of sensitivity, specificity, and accuracy for early DR detection and diagnosis (after detecting and removing blood vessels from the retinal images in abnormal cases, the abnormal lesions [18] will be clearly visible for DR diagnosis), while being compared with other state of the art in Tables 5 and 6.

The MATLAB R2011 version is used in this paper for the detection of blood vessels through the detection and elimination of OD.

## 6. Conclusion

The computer aided automatic detection and segmentation system for retinal blood vessels has been proposed in this paper. The segmentation of blood vessels was essential for DR severity classifications and diagnosis. This paper proposed a methodology which automatically detected and segmented the retinal blood vessels by eliminating the OD region from the retinal image in order to increase the segmentation accuracy level for blood vessel segmentation. The performance of the proposed segmentation methodology was analyzed with respect to ground truth images in two different publicly available datasets, consisting of normal and abnormal retinal images. The proposed system achieved the average vessel segmentation accuracy of 98.08% in DRIVE dataset and 95.94% in STARE dataset, respectively, with their corresponding ground truth images.

## Figures and Tables

**Table 1:** Anisotropic 3 × 3 diffusion mask.

*P*_NW_	*P*_N_	*P*_NE_
*P*_W_	*P*	*P*_E_
*P*_SW_	*P*_S_	*P*_SE_

**Table 2:** The directional filter coefficients.

Filter coefficient	Template values
*c*_1_	$\left[\begin{array}{ccc} 0 & 1 & 0\\ 0 & -1 & 0\\ 0 & 0 & 0 \end{array}\right]$
*c*_2_	$\left[\begin{array}{ccc} 0 & 0 & 0\\ 0 & -1 & 0\\ 0 & 1 & 0 \end{array}\right]$
*c*_3_	$\left[\begin{array}{ccc} 0 & 0 & 0\\ 0 & -1 & 1\\ 0 & 0 & 0 \end{array}\right]$
*c*_4_	$\left[\begin{array}{ccc} 0 & 0 & 0\\ 1 & -1 & 0\\ 0 & 0 & 0 \end{array}\right]$
*c*_5_	$\left[\begin{array}{ccc} 0 & 0 & 1\\ 0 & -1 & 0\\ 0 & 0 & 0 \end{array}\right]$
*c*_6_	$\left[\begin{array}{ccc} 0 & 0 & 0\\ 0 & -1 & 0\\ 0 & 0 & 1 \end{array}\right]$
*c*_7_	$\left[\begin{array}{ccc} 0 & 0 & 0\\ 0 & -1 & 0\\ 1 & 0 & 0 \end{array}\right]$
*c*_8_	$\left[\begin{array}{ccc} 1 & 0 & 0\\ 0 & -1 & 0\\ 0 & 0 & 0 \end{array}\right]$

**Table 3:** Evaluation results in DRIVE dataset.

Image sequence	DRIVE dataset (normal)			DRIVE dataset (abnormal)		
	Se	Sp	Acc	Se	Sp	Acc
1	96.2	99.1	98.9	93.1	98.3	96.7
2	95	99	98.3	95.3	99.1	97.2
3	94.6	99.1	98.2	94.7	99.3	99.6
4	92.8	97.9	96.2	93.8	98	98.2
5	95.6	99.5	97.7	94.3	96.5	97.1
6	95	98.9	99.1	93.1	99.6	98.6
7	94.1	98.4	98.7	93.1	98.5	99.1
8	94	96.1	96.6	—	—	—
9	93.4	99.9	99.1	—	—	—
10	92.8	98.2	98.6	—	—	—
11	96.5	99.5	99.4	—	—	—
12	94.7	98.6	98.8	—	—	—
13	94.9	99.5	98.7	—	—	—
14	93.4	97.9	99.7	—	—	—
15	92.6	98.7	98.1	—	—	—
16	94.1	98.6	97.1	—	—	—
17	93.8	98.9	97.8	—	—	—
18	91.1	99.2	96.5	—	—	—
19	90	96.1	98.2	—	—	—
20	91.4	98.7	97.9	—	—	—
**21**	92.6	97.8	98.5	—	—	—
**22**	93.4	98.1	98.1	—	—	—
**23**	94	97.3	97.2	—	—	—
**24**	91.1	98.1	98.7	—	—	—
**25**	90.1	96.2	98.1	—	—	—
**26**	92.1	97.8	98.9	—	—	—
**27**	95.2	98.4	98.5	—	—	—
**28**	96.1	98.2	98.4	—	—	—
**29**	95.1	97.9	99.2	—	—	—
**30**	96.2	97.9	98.5	—	—	—
**31**	98.1	97.6	96.1	—	—	—
**32**	96.6	97.1	97.4	—	—	—
**33**	98.1	99.1	96.2	—	—	—

Average	94.08	98.28	98.10	93.91	98.47	98.07

“—” indicates that there is no data available.

**Table 4:** Evaluation results in STARE dataset.

Image sequence	STARE dataset (normal)			Image sequence	STARE dataset (abnormal)		
	Se	Sp	Acc		Se	Sp	Acc
1	92.5	98.4	94.5	1	93.8	98.6	94.9
2	93.7	99.6	95.7	2	94.4	99.4	95.3
3	93.8	99.4	96	3	95.1	99.6	96.4
4	94.9	98.5	96	4	95.6	98.3	95.6
5	92.1	96.9	95.7	5	93.4	96.8	96.1
6	93.1	99.8	96.1	6	93.8	99.9	95.7
7	92	98.5	94.4	7	93.3	98.8	94.8
8	93.3	99.7	96.6	8	94	99.4	96.2
9	92.8	99.2	96.7	9	94.1	99.3	97.1
10	92.8	99.5	97.7	10	93.5	99.7	97.3

Average	93.1	98.95	95.94	Average	94.1	98.98	95.94

**Table 5:** Performance comparisons on DRIVE dataset.

Algorithm	Sensitivity (%)	Specificity (%)	Accuracy (%)
Proposed method^*^	93.99	98.37	98.08
Fraz et al. [7] (2012)	74.06	98.07	94.8
Xiao et al. [8] (2013)	75.13	97.92	95.29
Manoj et al. [9] (2013)	94.29	98.75	96.23
Bansal and Dutta [10] (2013)	86.53	98.33	97.28
Marín et al. [5] (2011)	70.67	98.01	94.52
Mendonça and Campilho [6] (2006)	⋯	⋯	96.33
Palomera-Pérez et al. [4] (2010)	⋯	⋯	92.5
Budai et al. [11] (2013)	64.40	98.70	95.72

^*^The methodologies compared with proposed method did not consider OD elimination before vessel segmentation.

**Table 6:** Performance comparisons in STARE dataset.

Algorithm	Sensitivity (%)	Specificity (%)	Accuracy (%)
Proposed method^*^	93.60	98.96	95.94
Fraz et al. [7] (2012)	72.62	97.64	95.11
Xiao et al. [8] (2013)	71.47	97.35	94.76
Manoj et al. [9] (2013)	93.14	98.84	95.83
Marín et al. [5] (2011)	69.44	98.19	95.26
Mendonça and Campilho [6] (2006)	⋯	⋯	95.79
Palomera-Pérez et al. [4] (2010)	⋯	⋯	92.6
Budai et al. [11] (2013)	58.00	98.20	93.86

^*^The methodologies compared with proposed method did not consider OD elimination before vessel segmentation.

**Table 7:** Comparison of average blood vessel computational time (in sec).

Algorithm	Average time taken for a single retinal image (blood vessel segmentation) (seconds)	
	STARE	DRIVE
Proposed	1.27	1.010
Marín et al. [5] (2011)	90.0	90.0
Budai et al. [11] (2013)	1.310	1.040
Soares et al. [13] (2006)	180.0	180.0
Frangi et al. [17] (1998)	1.620	1.270
